# Extension of multifactor dimensionality reduction for identifying multilocus effects in the GAW14 simulated data

**DOI:** 10.1186/1471-2156-6-S1-S145

**Published:** 2005-12-30

**Authors:** Hao Mei, Deqiong Ma, Allison Ashley-Koch, Eden R Martin

**Affiliations:** 1 Center for Human Genetics, Duke University Medical Center, Durham, NC 27710 USA; 2 North Carolina State University, Bioinformatics Center, Raleigh, NC 27606 USA

## Abstract

The multifactor dimensionality reduction (MDR) is a model-free approach that can identify gene × gene or gene × environment effects in a case-control study. Here we explore several modifications of the MDR method. We extended MDR to provide model selection without crossvalidation, and use a chi-square statistic as an alternative to prediction error (PE). We also modified the permutation test to provide different levels of stringency. The extended MDR (EMDR) includes three permutation tests (fixed, non-fixed, and omnibus) to obtain *p*-values of multilocus models. The goal of this study was to compare the different approaches implemented in the EMDR method and evaluate the ability to identify genetic effects in the Genetic Analysis Workshop 14 simulated data. We used three replicates from the simulated family data, generating matched pairs from family triads. The results showed: 1) chi-square and PE statistics give nearly consistent results; 2) results of EMDR without cross-validation matched that of EMDR with 10-fold cross-validation; 3) the fixed permutation test reports false-positive results in data from loci unrelated to the disease, but the non-fixed and omnibus permutation tests perform well in preventing false positives, with the omnibus test being the most conservative. We conclude that the non-cross-validation test can provide accurate results with the advantage of high efficiency compared to 10-cross-validation, and the non-fixed permutation test provides a good compromise between power and false-positive rate.

## Background

Gene × gene and gene × environment interactions undoubtedly play an important role in risk of complex diseases. These interactive effects, particularly when there are weak marginal effects, may be difficult to detect with traditional analysis approaches. Though classic statistical methods (e.g., logistical regression) are commonly used, as the number of possible interactions increases, the number of interaction terms grows exponentially with the addition of the main effect of each gene, leading to over-parameterization and low power in models with high-dimensionality [1]. To address this concern, the multifactor dimensionality reduction (MDR) was developed to identify interactions among multiple factors, which together influence disease susceptibility [2].

The MDR method was inspired by the combinatorial partitioning (CP) method, which builds models using data-driven methods [3]. In contrast to CP, MDR always reduces the dimensionality to two partitions, high risk and low risk. By applying the technique of n-1 crossvalidation (keeping n-1 groups for training and leaving out one group for validation), MDR identifies the best model with maximum consistency and minimum prediction error. To evaluate whether the best model is statistically significant, a permutation test based on data simulation is applied to obtain a *p*-value. Though the MDR has been shown to provide a powerful approach [4], it does have some limitations. First, the MDR uses prediction error as an estimate of the internal validity of the selected model. Prediction error represents the percentage of misclassification error in the test dataset. Theoretically, the smaller the prediction error is, the better the model is at predicting disease status. However, in real data analysis of complex diseases, significant models often have relatively high prediction error, typically greater than 40%. This is a particular problem when risk alleles are common. A second difficulty is that the MDR ideally determines the best model with maximum consistency and minimum prediction error. But in real analysis, consistency and prediction error often conflict. We have experienced this in our analysis of real data, where, using 10-fold cross-validation, a model with consistency as high as 9 may have prediction error much higher than a model with consistency of only 1. Finally, the MDR permutation test simultaneously assesses significance over all combinations of marker loci evaluated (i.e., complete permutation test, discussed below), which is powerful in decreasing type I error, but can lead to a severe loss in power.

To address these limitations, we have extended the MDR in several ways (EMDR, as we refer to it). The EMDR provides both a chi-square statistic and prediction error with or without 10-fold cross-validation for selection of the best model. Three permutation tests are provided to obtain the *p*-value, based on different hypotheses. We used the Genetic Analysis Workshop 14 (GAW14) simulated data, specifically targeting regions with known interactions (based on knowledge of the answers), to evaluate the novel features of EMDR and compare it with the original MDR.

## Methods

### Dataset

The dataset used for validation of the EMDR was the simulated GAW14 data of Kofendrerd Personality Disorder (KPD). The broadest phenotype, P3, was selected for this study. We selected the first qualifying family triad (2 parents and 1 affected offspring) from each pedigree in the simulated family dataset. To provide an adequate sample size we pooled replicates 1–3 for a total of 440 independent triads.

We consulted the answers to target our exploration of multilocus effects to specific loci. The answers revealed that there are 2-way interactions involving 4 loci affecting risk for P3 (D1–D4 and D2–D3). We considered several sets of markers (Table 1) to explore the properties of the EMDR. Due to computational limitations, we restricted the size of each marker set to 8 markers. Data I contain 8 independent markers unlinked to any of the disease loci on chromosome 2. In Data II, 8 markers were selected surrounding disease loci D1 and D4. Similarly, 8 markers in Data III surround disease loci D2 and D3 (Table 1).

### Statistics

The EMDR, like MDR, develops a locus model to predict affection status, grouping genotypes into high- and low-risk classes. The 10-fold cross-validation test of EMDR divides the dataset into 10 training datasets (each with 90% of the sample), which are used to train the locus model, and 10 test datasets (each with the remaining 10% of the sample), which are used to validate the locus model [5]. Ten-fold cross-validation can output at most 10 different locus models from which the best model is selected based on statistics computed in the test dataset. Two statistics are computed for each model: chi-square and prediction error (PE). PE is calculated as the proportion of cases and controls that are misclassified by the model. The chi-square statistic measures the association between genotype (high-risk and low-risk group) and affection status (case and control group) in a two-way table. It is calculated as sum of the square of the differences between the observed and expected frequency in each cell, divided by the expected value, across all of the cells in the table: . The identification of the best model is based on the value of chi-square or PE. It is possible in a 10-cross-validation EMDR run to generate two different best models in terms of largest chi-square or smallest PE.

Assessing statistical significance of a model depends on a *p*-value from a permutation test. A model with a *p*-value < 0.05 is regarded as a significant multilocus effect in our analyses. EMDR provides three types of permutation tests in which each adjusts for the data reduction technique across locus combinations to a different extent. All permutation tests hypothesize that a specific *n*-locus genotype model is independent of disease status. To identify the locus model, matched pairs from the GAW14 simulated data were constructed by deriving transmitted and nontransmitted alleles from independent family triads (parents and an affected child), where the case genotype is the transmitted pair of alleles and the pseudocontrol is the nontransmitted pair. The simulated data for the permutation test are generated by permuting the status of case and control within each pair (e.g., for a family triad transmitted and nontransmitted genotypes are permuted randomly). The fixed permutation test considers only the specific best *n*-locus model (e.g., suppose loci 1 and 2 are selected for the 2-locus model). The exact same set of loci is then evaluated in the permuted dataset, redefining high- and low-risk genotype classes and recomputing the statistic (PE, chi-square) for that model. After conducting the procedure a large number of times (e.g., 1,000 times), we compared the observed statistic to the distribution of permuted statistics to obtain the *p*-value.

The non-fixed and the omnibus permutation tests are more computationally intensive. Suppose the total number of markers or loci in dataset is *m*. To compute the *p*-value of a specific *n*-locus model, the non-fixed permutation test computes the statistic in the permuted data considering all possible *n*-locus models (i.e., all m!/ [(m-n)!*n!] models). In the omnibus permutation test, the statistic is selected from the entire set of models, i.e., all 1-locus, 2-locus, ..., *k*-locus models (i.e., all  models). For bi-allelic loci, the value of *k*, the largest number allowed for testing, follows the formula: j/3^k^>3 (j is the size of the test dataset in 10-fold cross-validation).

## Results

### Unlinked loci

Data I (markers unlinked to disease loci) was first analyzed by the EMDR to evaluate false-positive rates. For non-cross-validation, the non-fixed permutation test did not detect any significant models (Table 2). In contrast, the fixed permutation test yielded significant 2-locus models (*p *= 0.05 and 0.02 for chi-square and PE, respectively). The results are similar to 10-fold cross-validation. As expected, the omnibus test did not give us the significant results (data not shown).

### D1–D4 multi-locus effects

Data II includes a set of markers flanking loci D1 and D4. Ten-fold cross-validation and non-cross-validation identified the same best 1-, 2-, and 3-locus models using both chi-square and PE test statistics (Table 3). However, the two markers involved in the best 2-locus model are all located in D4 region. Only the best 3-locus model included markers near both D1 and D4. This effect was identified by all permutation tests except non-fixed permutation test with 10-fold cross-validation.

### D2–D3 multi-locus effects

Data III includes markers flanking D2 and D3. Both the chi-square and PE statistics gave the same 1- and 2-locus models with and without cross-validation (Table 4). However, the chi-square statistic displayed a different 3-locus model from PE. This discrepancy is likely due to computational differences of the statistics when there are minor differences between the models. All best models were significant under fixed and non-fixed permutation test. However, neither of 2- and 3-locus models include markers around D2. The omnibus permutation test gave a significant 3-locus model only.

Table 5 shows models other than the best model that were also significant with the EMDR. The chi-square and PE under fixed and non-fixed permutation test detected significant 2- and 3-locus models that include markers around both D2 and D3. This shows that although the D2–D3 effect was not detected as the best model, it was successfully identified among significant models.

## Discussion

Our analysis of the GAW14 simulated data gives a comparison of the traditional MDR and several different options in the EMDR with the benefit of knowing the answers. The traditional MDR identifies the best marker models by 10-fold cross-validation using the PE statistic and obtains *p*-values by permutation testing (i.e., omnibus permutation in EMDR). Our EMDR extended the MDR to include a chi-square statistic in addition to PE, options for 10-fold cross-validation or non-cross-validation (with PE and chi-square statistics), and multiple permutation tests (fixed, non-fixed, and omnibus). By comparing these different methods, we found that 10-fold cross-validation and non-cross-validation were fairly consistent in identification of the best locus-model. The fixed permutation test produced several false-positive results, and the omnibus permutation test of the traditional MDR lost the power to identify the known D1–D4 and D2–D3 interaction. In Data I (unlinked to disease loci), non-cross-validation with non-fixed permutation test generated *p*-values over 0.5 for nearly for all of the best models (1-, 2- and 3- models) with no false positives. Non-cross-validation with the non-fixed permutation test also correctly detected significant marker effects between D1–D4 in Data II and significant marker effects between D2–D3 in Data III, suggesting that non-cross-validation with the non-fixed permutation test has the power to identify true multilocus interactions (D1–D4 and D2–D3 interactions). We conclude that non-cross-validation using the non-fixed permutation test performs well on matched data from three replicates (440 case/control pairs) from the GAW14 simulated data.

In the GAW14 simulated data, we found that the model statistics of the 10-fold cross-validation approach had large variation, leading to inconsistent conclusions. Small sample size of the test data and genetic heterogeneity could cause these inconsistencies, under which non-cross-validation performs better. Non-cross-validation improved two limitations of the original MDR (computational intensity and high dimensionality with small sample) [6]. Non-cross-validation has the advantage of high computational efficiency (no validation by test data is needed), no false-positive results, and was more consistently able to detect true loci compared with the original 10-fold cross-validation approach of the MDR.

Similar to MDR, the EMDR has the power to detect joint effects of multiple genes on disease risk. However, the method cannot itself differentiate interactions from main effects, nor can it distinguish whether a joint effect is driven by a strong marginal effect. For example, in the Data II analysis, EMDR identified (6), (2 6), and (2 6 7) as significant best models, however, it is hard to tell if the gene × gene effect within (2 6) and (2 6 7) is driven by (6) only or due to the interaction between D2 and D3. One possible solution is to use logistic regression to model the genotype effects. We tested (2 6) model in this study, but found no interaction between locus 2 and 6 while forcing all factors into the model, suggesting that the 2-locus model might be due to the combination effect of independent main effects. Interpretation of models developed by the EMDR in complex genetic diseases is an important direction for future studies.

## Abbreviations

CP: Combinatorial partitioning

EMDR: Extended MDR

GAW14: Genetic Analysis Workshop 14

KPD: Kofendrerd personality disorder

MDR: Multifactor dimensionality reduction

PE: Prediction error

## Figures and Tables

**Table 1:** Marker description of data I, II and III

	Data I	Data II		Data III	
			
Marker Index	Marker Name	Marker Name	Loci	Marker Name	Loci
1	C02R0092	**C01R0052**^**a**^	**D1**	B03T3064	
2	C02R0105	**B01T0561**	**D1**	B03T3065	
3	C02R0118	B01T0562		B03T3066	
4	C02R0131	B01T0563		**B03T3067**	**D2**
5	C02R0144	B09T8335		B05T4135	
6	C02R0157	**C09R0765**	**D4**	**B05T4136**	**D3**
7	C02R0170	**B09T8337**	**D4**	**C05R0380**	**D3**
8	C02R0183	B09T8338		B05T4138	

^a ^Bold text indicate that *p*-values of the statistics are significant at alpha = 0.05.

**Table 2:** non-crossvalidation analysis of data I

		*p*-Value of chi square			*p*-Value of prediction error	
				
Model	*χ*^2^	Fixed permutation	Non-fixed permutation	Prediction error	Fixed permutation	Non-fixed permutation
(8)	1.939	0.284	>0.284	0.4773	0.198	>0.198
(4 8)	7.099	**0.05**^**a**^	0.592	0.4557	**0.024**	0.363
(2 7 8)	12.49	0.228	>0.228	0.4409	0.207	>0.207

^a ^Bold text indicate that markers flanking or located at simulated disease loci.

**Table 3:** Analysis of data II

		*p*-Value of chi square			*p*-Value of prediction error		
				
Marker	*χ*^2^	Fixed permutation	Non-fixed permutation	Prediction error of test data	Fixed permutation	Non-fixed permutation	Omnibus permutation
10-fold cross-validation							
(6)	1.446	0.136	0.572	0.462	**0.018**^**a**^	0.13	0.184
(6 7)	3.101	**0.002**	**0.046**	0.462	**0**	**0.01**	0.184
(2 3 8)	2.284	**0.027**	0.094	0.432	**0**	**0.008**	**0.043**
Non-cross-validation							
(6)	6.630	**0.03**	0.186	0.458	**0.017**	0.138	
(6 7)	20.985	**0**	**0.006**	0.430	**0**	**0.019**	
(2 3 8)	39.324	**0**	**0.005**	0.394	**0**	**0.003**	

^a ^Bold text indicate that *p*-values of the statistics are significant at alpha = 0.05.

**Table 4:** Analysis of data III

		*p*-Value of chi square			*p*-Value of prediction error		
				
Marker	*χ*^2^	Fixed permutation	Non-fixed permutation	Prediction error of test data	Fixed permutation	Non-fixed permutation	Omnibus permutation
10-fold crossvalidation							
(6)	2.507	**0.006**^**b**^	0.088	0.444	**0.002**	**0.006**	0.255
(6 7)	3.647	**0.002**	**0.016**	0.422	**0**	**0**	0.117
(5 6 7)	**--**^**a**^	**--**	**--**	0.398	**0**	**0**	**0.042**
(6 7 8)	4.782	**0**	**0.004**	**--**	**--**	**--**	**--**
Non crossvalidation							
(6)	13.870	**0.001**	**0.006**	0.438	**0.001**	**0.004**	
(6 7)	35.276	**0**	**0**	0.408	**0**	**0.002**	
(5 6 7)	**--**	**--**	**--**	0.384	**0**	**0**	
(6 7 8)	49.657	**0**	**0**	**--**	**--**	**--**	

^a ^**--**, not tested

^b ^Bold text indicate that *p*-values of the statistics are significant at alpha = 0.05.

**Table 5:** D2–D3 effect of data III

		*p*-value of chi-square		*p*-value of prediction error		
			
Model	Effect	non-cross-validation non-fixed permutation	10-fold cross-validation, non-fixed permutation	non-cross-validation, non-fixed permutation	10-fold cross-validation, non-fixed permutation	10-fold cross-validation, omnibus permutation
(2 6)	D2–D3	0	0.02	0.002	0	0.122
(2 6 7)	D2–D3	0	0.028	0	0.002	0.09
